# Misinformation or education? A comparative analysis of Instagram posts related to full mouth rehabilitation

**DOI:** 10.3389/fdmed.2025.1585575

**Published:** 2025-07-24

**Authors:** Amal Alfaraj, Faris A. Alshahrani, Meshal Albalawi, Naif AlQahtani, Mohammed Alhajji, Ali Sulaiman Alharbi, Sultan Ainoosah, Khalifa S. Al-Khalifa

**Affiliations:** ^1^ Department of Prosthodontics and Dental Implantology, College of Dentistry, King Faisal University, Al-Ahsa, Saudi Arabia; ^2^ Department of Substitutive Dental Sciences, College of Dentistry, Imam Abdulrahman Bin Faisal University, Dammam, Saudi Arabia; ^3^ College of Dentistry, Imam Abdulrahman Bin Faisal University, Dammam, Saudi Arabia; ^4^ Department of Substitutive Dental Science, College of Dentistry, Taibah University, Madinah, Saudi Arabia; ^5^ Department of Preventive Dental Sciences, College of Dentistry, Imam Abdulrahman Bin Faisal University, Dammam, Saudi Arabia

**Keywords:** full mouth rehabilitation, Instagram health education, digital misinformation, social media dentistry, public engagement

## Abstract

**Introduction:**

Social media platforms have become pivotal in health communication, with Instagram serving as a key channel for sharing medical and dental information. Full Mouth Rehabilitation (FMR), a complex treatment aimed at restoring oral function and aesthetics, remains underrecognized by the general public. This study investigates the nature and accuracy of Instagram content related to FMR.

**Methods:**

A cross-sectional content analysis was conducted on 144 Instagram posts using FMR-related hashtags. Posts were classified based on content type, poster role (e.g., dental professional, clinic, influencer), engagement metrics (likes, comments), and accuracy of information. Statistical tests were applied to assess variations across content categories.

**Results:**

Marketing-oriented content constituted the majority of posts (75.7%), while educational content accounted for only 9%. Most posts (86.8%) contained non-factual or misleading information. In contrast, all educational posts were factually accurate. Engagement levels did not significantly differ between factual and non-factual posts. Dental professionals were responsible for only 5.6% of the total content, reflecting a notable lack of expert presence.

**Discussion:**

Instagram holds significant potential for disseminating accurate dental health information. However, the dominance of promotional content and the low involvement of dental professionals contribute to a high prevalence of misinformation. To enhance the platform's reliability as a health communication tool, increased participation by dental experts and improved content oversight are essential. Future research should evaluate strategies for promoting evidence-based information on social media, particularly for advanced procedures like FMR.

## Introduction

Full Mouth Rehabilitation (FMR) is a comprehensive dental treatment aimed at restoring oral function, aesthetics, and overall oral health for patients with complex dental conditions, such as extensive tooth wear, edentulism, or malocclusion (1). This transformative approach often requires a multidisciplinary team that integrates expertise from prosthodontics, orthodontics, periodontics, oral surgery, and other specialties, depending on the complexity of each case (2). FMR not only resolves functional impairments and aesthetic concerns but also contributes to long-term oral health and improved self-confidence, making it a cornerstone of advanced dental care (3).

Despite its clinical benefits, public awareness of FMR remains limited. Several barriers, such as the high cost of treatment, limited availability of specialists, and misinformation about advanced dental procedures, restrict its broader adoption (4). A growing body of research indicates that social media platforms, particularly Instagram, play an increasing role in shaping public perceptions of healthcare services, including dentistry (5). However, while these platforms serve as valuable educational tools, they also pose risks of spreading misleading or promotional content that may not always align with evidence-based dental practices.

Social media, particularly Instagram, has emerged as a dominant force in digital health communication (6). With its visually driven format and algorithmic content promotion, Instagram enables dental professionals to showcase FMR outcomes, engage with patients, and contribute to public health education (5). However, social media algorithms prioritize engagement metrics such as likes, shares, and comments, often amplifying promotional content over factual educational materials (7). This creates an environment where commercial interests may overshadow accurate health communication, potentially influencing patient decision-making based on incomplete or misleading information.

Recent studies have highlighted the proliferation of health misinformation on social media, particularly within cosmetic and elective medical fields, including dentistry (8). The accessibility of user-generated content has led to a diversification of voices in digital health communication, yet professional oversight and accuracy verification remain limited. Despite increasing patient reliance on Instagram for healthcare information, dental professionals remain underrepresented in digital discourse, limiting the availability of expert-driven, evidence-based content.

Given the growing impact of social media on healthcare education, it is critical to evaluate the role of Instagram in disseminating FMR-related information. This study aimed to describe the characteristics and assess the informational accuracy of Instagram posts related to Full Mouth Rehabilitation (e.g., clinicians, dental practices, influencers). A key research question is whether Instagram serves as an effective public health communication tool for FMR or if misinformation and commercial promotion undermine its educational potential. The hypothesis suggests that while Instagram provides a platform for information-sharing, its content quality and reliability vary significantly, potentially affecting public perception and decision-making regarding FMR treatment.

## Methods

**Figure 1:**
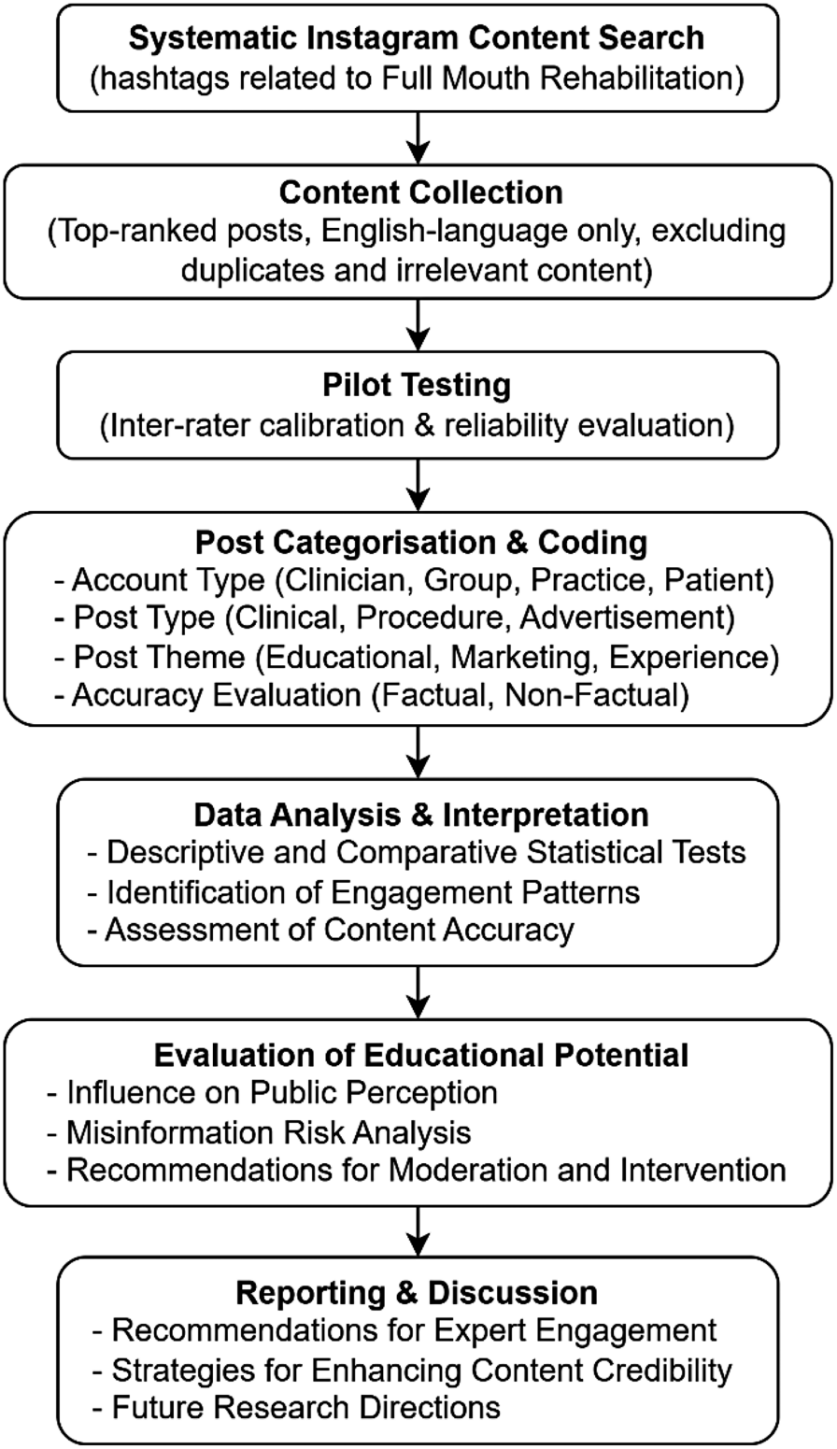
Flow chart of analyzing full mouth rehabilitation related hashtags.

Given the increasing reliance on Instagram as a source of health information, this study sought to evaluate the role of social media in shaping public perceptions and potential misinformation regarding FMR. The study design is illustrated in Figure 1.

The study focused on English-language content and was based on a systematic search of Instagram posts tagged with relevant hashtags. The methodology, including data collection and analysis, was adapted from prior research on social media trends in healthcare (9, 10). Hashtags were selected based on their popularity, relevance to FMR, and engagement metrics on Instagram. The final set of 12 hashtags included the following hashtags: #fullmouthrehabilitation, #oralrehabilitation, #fullmouthreconstruction, #fullmouthrehab, #fullmouthrestoration, #mouthrehabilitation, #fullmouthrejuvenation, #mouthreconstruction, #fullmouthrestorations, #oralreconstruction, #Oralrehab and #mouthrehab.

To standardize data collection and minimize algorithmic bias, a pilot test was conducted using a small sample of posts. Discrepancies in categorization were resolved through discussion and consensus among researchers to ensure uniform evaluation criteria. Two coders conducted a pilot test by coding 50 random posts related to dental smile alterations and FMR. A weighted Kappa was utilized to test the reliability. Both coders classified another 50 randomly selected posts and were reclassified one week after the first coding. The weighted Kappa was 0.775 for inter-agreement and 0.820 and 0.720 for intra-agreement (two coders) with an overall excellent reliability. Any conflict in coding was resolved by a gold standard examiner via discussion to ensure full understanding of post categorization.

Data were collected on a single day to control for Instagram's dynamic content ranking system, which prioritizes engagement metrics such as likes, shares, and comments. For each hashtag, the top 12 ranked posts were retrieved following Instagram's search and discovery algorithm. Posts deemed irrelevant or duplicate were excluded.

### Data extraction and coding

Each post was categorized based on the following variables: Account type (identified as a dental practice, dental professional, dental laboratory, influencer, or commercial entity), post type (classified into clinical procedure, practice advertisement, product promotion, or patient experience), poster role (differentiated as a dental organization, company, patient, or licensed dentist), post theme (categorized as educational, marketing, or experience sharing), posts were further classified based on intent (educational vs. promotional) and content format (image, video, reel, or carousel). Engagement metrics, including likes, comments, follower counts, and hashtags used, were recorded for each post.

### Accuracy of claims and misinformation identification

To assess the accuracy of claims, posts were evaluated using a binary classification scale (factual vs. non-factual). This was adapted from previous methodologies in health misinformation research (10). A post was classified as factual if its content aligned with peer-reviewed literature, clinical guidelines, or professional dental sources. Non-factual claims included misleading statements, exaggerated treatment outcomes, or incorrect procedural descriptions.

To ensure reliability and minimize evaluator bias, data coding was conducted independently by two trained researchers. A calibration process was implemented during the pilot phase to establish coding consistency, reducing inter-evaluator variability.

### Statistical analysis

Descriptive statistics, including means, standard deviations, frequencies, and percentages, were used to summarize data distributions. Normality testing using the Shapiro–Wilk test indicated that the data were not normally distributed, necessitating the use of non-parametric statistical methods.

The Mann–Whitney *U* test was employed for comparisons between two groups (e.g., educational vs. promotional posts). While the Kruskal–Wallis test was applied for comparisons across multiple groups (e.g., account types, post themes). Furthermore, pairwise comparisons were adjusted using the Bonferroni method to reduce the likelihood of Type I errors. In addition, categorical variables (e.g., accuracy of claims) were analyzed using the chi-square test, with Monte Carlo corrections applied when cell counts were below five. All statistical analyses were performed using IBM SPSS Statistics (version 22.0), with a significance threshold of *p* < 0.05.

### Ethical considerations

This study adhered to ethical guidelines for digital research and the analysis of publicly available social media content. As all posts were publicly accessible, no direct interaction with users occurred, and no identifiable private data were collected. Given that the study did not involve human participants, institutional review board (IRB) approval was not required. However, ethical best practices for social media research were followed, including transparency in methodology and adherence to data privacy guidelines.

## Results

### Background characteristics of Instagram posts

Table 1 presents the general characteristics of 144 Instagram posts related to Full Mouth Rehabilitation (FMR). Photos were the most common content format (50%), followed by reels and carousels (22.2% each), and videos (7.6%). Most posts were in English (75%), and 56.9% lacked a written description.

**Table 1:** Characteristics of posts under full mouth rehabilitation related hashtags (*n* = 144).

Variable	Quantitative characteristics	
No. of likes	Mean ± SD	1,214.95 ± 8,817.865
No. of comments	Mean ± SD	12.81 ± 34.460
No. of followers	Mean ± SD	1,37,224.43 ± 1,97,241.114
No. of #	Mean ± SD	15.77 ± 8.835
Variable	Quantitative characteristics	*n* (%)
# linked:	Related	82 (56.9)
	Not related	62 (43.1)
*First 10 comments: (*n* = 104)	Related	93 (89.4)
	Not related	11 (10.6)
*Are there fake comments? (*n* = 104)	No	104 (100.0)
	Yes	0 (0.0)
*Does the account interact with the comments? (*n* = 104)	Yes	58 (55.8)
	No	46 (44.2)
Language:	English	108 (75.0)
	Other	36 (25.0)
is there a description of the post?	Yes	62 (43.1)
	No	82 (56.9)
Did the owner raised an argument or asked a question?	Yes	11 (7.6)
	No	133 (92.4)
Content type:	Photo	69 (50.0)
	Video	11 (7.6)
	Carousel	32 (22.2)
	Reel	32 (22.2)
Poster role:	Patient	86 (59.7)
	Dental related group	50 (34.7)
	Dentist	8 (5.6)
Post theme:	Marketing	109 (75.7)
	Sharing experience	22 (15.3)
	Informative/ educational	13 (9.0)
Post type:	Clinical case	101 (70.1)
	Concern or procedure discussion	13 (9.1)
	Practice advertisement	30 (20.8)
Account type:	Clinician	69 (47.9)
	Dental interested group	30 (20.8)
	Practice	45 (31.3)
Accuracy of claims:	Facts	19 (13.2)
	Non-facts	125 (86.8)
Does the post as a watermark?	Yes	85 (59.0)
	No	59 (41.0)

SD, standard deviation (*n* = 144), except * *n* = 10.

The average number of likes per post was 1,214.95 (±8,817.87), with 12.81 (±34.46) comments and a mean follower count of 137,224.43 (±197,241.11). Among posts that received comments (*n* = 104), 89.4% of the first 10 comments were related to the content, with no fake comments identified. Account interaction with commenters was observed in 55.8% of cases.

Patients posted the majority of content (59.7%), followed by dental-related groups (34.7%) and dentists (5.6%). Most posts were marketing in nature (75.7%), while only 9% were categorized as educational. In terms of the accuracy of the information, 86.8% of posts contained non-factual claims—such as unrealistic promises of “permanent whitening with FMR” or “instant bite correction”—while all educational posts were factually accurate. Watermarks were found in 59.0% of posts.

### Post themes

Table 2 shows post characteristics categorized by theme: marketing, experience sharing, and educational. While marketing posts had the highest average number of likes, the differences across themes were not statistically significant. Educational posts had the highest average follower count, while experience-sharing posts received the most comments.

**Table 2:** Comparison of post characteristics according to post theme.

Variable	Quantitative metrics	Marketing	Sharing experience	Informative/ educational	*P*
Number of likes	Mean ± SD	1,454.29 ± 10,110.788	553.05 ± 1,509.951	328.31 ± 600.088	0.847
Number of followers	Mean ± SD	1,32,663.71 ± 1,89,623.597	1,41,456.45 ± 2,00,874.732	1,68,302.46 ± 2,60,980.749	0.842
Number of comments	Mean ± SD	13.19 ± 36.482	13.91 ± 33.796	7.69 ± 11.912	0.853
Number of #	Mean ± SD	16.22 ± 9.238	14.32 ± 8.593	14.46 ± 5.027	0.563
**First 10 comments: *n* (%) (*n* = 104)	Related	76 (90.5)	11 (84.6)	6 (85.7)	0.215
	Not related	8 (9.5)	2 (15.4)	1 (14.3)	
**Fake comments: *n* (%) (*n* = 104)	Yes	0 (0.0)	0 (0.0)	0 (0.0)	0.069
	No	84 (100.0)	13 (100.0)	7 (100.0)	
**Interaction with the comments: *n* (%) (*n* = 104)	Yes	48 (57.2)	6 (46.2)	4 (57.1)	0.213
	No	36 (42.9)	7 (53.8)	3 (42.9)	
# linked: *n* (%)	Related	64 (58.7)	10 (45.4)	8 (61.6)	0.488
	Not related	45 (41.3)	12 (54.6)	5 (38.4)	
Language: *n* (%)	English	79 (72.5)	18 (81.8)	11 (84.6)	0.459
	other	30 (27.5)	4 (18.2)	2 (15.4)	
Post description: *n* (%)	Yes	50 (45.9)	6 (27.3)	6 (46.2)	0.276
	No	59 (54.1)	16 (72.7)	7 (53.8)	
Raised an argument or asked a question: *n* (%)	Yes	8 (7.3)	0 (0.0)	3 (23.1)	*0.044
	No	101 (92.7)	22 (100.0)	10 (76.9)	
Content type: *n* (%)	Photo	56 (51.4)	7 (31.8)	6 (46.2)	*0.038
	Video	7 (6.4)	4 (18.2)	0 (0.0)	
	Carousel	25 (22.9)	2 (9.1)	5 (38.4)	
	Reel	21 (19.3)	9 (40.9)	2 (15.4)	
Poster role: *n* (%)	Patient	67 (61.5)	16 (72.7)	3 (23.1)	*0.022
	Dental related group	38 (34.8)	4 (18.2)	8 (61.5)	
	Dentist	4 (3.7)	2 (9.1)	2 (15.4)	
Post type: *n* (%)	Clinical case	92 (84.4)	7 (31.8)	2 (15.4)	*<0.001
	Concern or procedure discussion	4 (3.7)	0 (0.0)	9 (69.2)	
	Practice advertisement	13 (11.9)	15 (68.2)	2 (15.4)	
Account type: *n* (%)	Clinician	55 (50.5)	8 (36.4)	6 (46.2)	0.521
	Dental interested group	24 (22.0)	4 (18.2)	2 (15.4)	
	Practice	30 (27.5)	10 (45.4)	5 (38.4)	
Accuracy of claims: *n* (%)	Facts	6 (5.5)	0 (0.0)	13 (100.0)	*<0.001
	Non-facts	103 (94.5)	22 (100.0)	0 (0.0)	
Watermark: *n* (%)	Yes	70 (64.2)	8 (36.4)	7 (53.8)	*0.049
	No	39 (35.8)	14 (63.6)	6 (46.2)	

SD, standard deviation.

* Significant at *p* < 0.05. (*n* = 144), except ** *n* = 104.

A significant difference was found in the accuracy of the information (*p* < 0.001): 100% of educational posts were factual, whereas 94.5% of marketing and all experience-sharing posts included non-factual content. Watermark presence also varied significantly (*p* = 0.049), being more frequent in marketing posts.

### Account types

Table 3 summarizes post characteristics based on the type of account: clinician, dental interest group, or dental practice. Although practices had the highest average number of likes, this was not statistically significant. Dental interest groups had significantly higher follower counts (*p* = 0.012) and used more hashtags (*p* = 0.001). Clinicians were more likely to interact with comments and use relevant hashtags.

**Table 3:** Comparison of post characteristics according to account type.

Variable	Quantitative metrics	Clinician	Dental interest group	Practice	*P*
Number of likes	Mean ± SD	454.46 ± 808.664	706.73 ± 786.501	2,719.84 ± 15,744.376	0.385
Number of followers	Mean ± SD	1,30,830.26 ± 1,96,138.514	2,24,678.97 ± 2,02,133.525	88,725.80 ± 1,79,918.069	*0.012
Number of comments	Mean ± SD	16.06 ± 43.406	10.37 ± 11.903	9.44 ± 28.671	0.554
Number of #	Mean ± SD	13.33 ± 7.798	20.43 ± 9.684	16.40 ± 8.564	*0.001
**First 10 comments: *n* (%) (*n* = 104)	Related	51 (92.7)	23 (95.8)	19 (76.0)	*0.006
	Not related	4 (7.3)	1 (4.2)	6 (24.0)	
**Fake comments: *n* (%) (*n* = 104)	Yes	0 (0.0)	0 (0.0)	0 (0.0)	*0.011
	No	55 (100.0)	24 (100.0)	25 (100.0)	
**Interaction with the comments: *n* (%) (*n* = 104)	Yes	36 (65.5)	11 (45.8)	11 (44.0)	*0.009
	No	19 (34.5)	13 (54.2)	14 (56.0)	
# linked: *n* (%)	Related	46 (66.7)	18 (60.0)	18 (40.0)	*0.018
	Not related	23 (33.3)	12 (40.0)	27 (60.0)	
Language: *n* (%)	English	42 (60.9)	26 (86.7)	40 (88.9)	*0.001
	other	27 (39.1)	4 (13.3)	5 (11.1)	
Post description: *n* (%)	Yes	33 (47.8)	18 (60.0)	11 (24.4)	*0.005
	No	36 (52.2)	12 (40.0)	34 (75.6)	
Raised an argument or asked a question: *n* (%)	Yes	5 (7.2)	2 (6.7)	4 (8.9)	0.925
	No	64 (92.8)	28 (93.3)	41 (91.1)	
Content type: *n* (%)	Photo	32 (46.4)	16 (53.3)	21 (46.6)	0.716
	Video	4 (5.8)	2 (6.7)	5 (11.1)	
	Carousel	19 (27.5)	6 (20.0)	7 (15.6)	
	Reel	14 (20.3)	6 (20.0)	12 (26.7)	
Poste theme: *n* (%)	Marketing	55 (79.7)	24 (80.0)	30 (66.7)	0.521
	Sharing experience	8 (11.6)	4 (13.3)	10 (22.2)	
	Informative/educational	6 (8.7)	2 (6.7)	5 (11.1)	
Poster role: *n* (%)	Patient	51 (73.9)	5 (16.7)	30 (66.7)	*<0.001
	Dental related group	12 (17.4)	24 (80.0)	14 (31.1)	
	Dentist	6 (8.7)	1 (3.3)	1 (2.2)	
Post type: *n* (%)	Clinical case	53 (76.8)	25 (83.4)	23 (51.1)	*<0.001
	Concern or procedure discussion	7 (10.2)	1 (3.3)	5 (11.1)	
	Practice advertisement	9 (13.0)	4 (13.3)	17 (37.8)	
Accuracy of claims: *n* (%)	Facts	10 (14.5)	2 (6.7)	7 (15.6)	*0.008
	Non-facts	59 (85.5)	28 (93.3)	38 (84.4)	
Watermark: *n* (%)	Yes	43 (62.3)	17 (56.7)	25 (55.6)	0.740
	No	26 (37.7)	13 (43.3)	20 (44.4)	

SD, standard deviation.

* Significant at *p* < 0.05. (*n* = 144), except ** *n* = 104.

Significant differences were also observed in language use and content description. Clinician accounts posted more frequently in languages other than English, while dental interest group posts were most likely to include descriptions. Accuracy of information varied (*p* = 0.008), with factual content appearing slightly more often in clinician and practice posts.

### Post types

Table 4 outlines characteristics based on post type: clinical cases, procedure discussions, and advertisements. Clinical case posts were the most interactive, receiving the highest number of comments and follower engagement, though differences were not statistically significant. However, they were also the least factual—93.1% contained misleading or exaggerated claims.

**Table 4:** Comparison of post characteristics according to post type.

Variable	Quantitative metrics	Clinical case	Concern or procedure discussion	Practice advertisement	*P*
Number of likes	Mean ± SD	570.35 ± 839.987	159.15 ± 269.602	3,842.63 ± 19,283.636	0.184
Number of followers	Mean ± SD	1,57,006.91 ± 1,98,416.669	1,19,365.38 ± 2,24,238.168	78,362.33 ± 1,73,737.312	0.150
Number of comments	Mean ± SD	14.51 ± 37.660	6.85 ± 11.950	9.63 ± 29.488	0.643
Number of #	Mean ± SD	16.25 ± 9.073	11.38 ± 6.021	16.07 ± 8.745	0.171
**First 10 comments: *n* (%) (*n* = 104)	Related	76 (90.5)	5 (83.3)	12 (85.7)	*<0.001
	Not related	8 (9.5)	1 (16.7)	2 (14.3)	
**Fake comments: *n* (%) (*n* = 104)	Yes	0 (0.0)	0 (0.0)	0 (0.0)	*<0.001
	No	84 (100.0)	6 (100.0)	14 (100.0)	
**Interaction with the comments: *n* (%) (*n* = 104)	Yes	47 (56.0)	3 (50.0)	8 (57.1)	*<0.001
	No	37 (44.0)	3 (50.0)	6 (42.9)	
# linked: *n* (%)	Related	64 (63.4)	9 (69.2)	9 (30.0)	*0.003
	Not related	37 (36.6)	4 (30.8)	21 (70.0)	
Language: *n* (%)	English	75 (74.3)	9 (69.2)	24 (80.0)	0.719
	other	26 (25.7)	4 (30.8)	6 (20.0)	
Post description: *n* (%)	Yes	48 (47.5)	4 (30.8)	10 (33.3)	0.249
	No	53 (52.5)	9 (69.2)	20 (66.7)	
Raised an argument or asked a question: *n* (%)	Yes	7 (6.9)	3 (23.1)	1 (3.3)	0.072
	No	94 (93.1)	10 (76.9)	29 (96.7)	
Content type: *n* (%)	Photo	57 (56.4)	6 (46.2)	6 (20.0)	*<0.001
	Video	7 (6.9)	0 (0.0)	4 (13.3)	
	Carousel	22 (21.8)	6 (46.2)	4 (13.3)	
	Reel	15 (14.9)	1 (7.6)	16 (53.4)	
Post theme: *n* (%)	Marketing	92 (91.1)	4 (30.8)	13 (43.3)	*<0.001
	Sharing experience	7 (6.9)	0 (0.0)	15 (50.0)	
	Informative/educational	2 (2.0)	9 (69.2)	2 (6.7)	
Poster role: *n* (%)	Patient	70 (69.3)	3 (23.1)	13 (43.3)	*<0.001
	Dental related group	31 (30.7)	8 (61.5)	11 (36.7)	
	Dentist	0 (0.0)	2 (15.4)	6 (20.0)	
Account type: *n* (%)	Clinician	53 (52.5)	7 (53.8)	9 (30.0)	*0.008
	Dental interest group	25 (24.7)	1 (7.7)	4 (13.3)	
	Practice	23 (22.8)	5 (38.5)	17 (56.7)	
Accuracy of claims: *n* (%)	Facts	7 (6.9)	10 (76.9)	2 (6.7)	*<0.001
	Non-facts	94 (93.1)	3 (23.1)	28 (93.3)	
Watermark: *n* (%)	Yes	64 (63.4)	7 (53.8)	14 (46.7)	0.243
	No	37 (36.6)	6 (46.2)	16 (53.3)	

SD, standard deviation.

* Significant at *p* < 0.05. (*n* = 144), except ** *n* = 104.

In contrast, procedure discussion posts had the highest proportion of accurate information (76.9%). Content type differed significantly by post type (*p* < 0.001), with photos dominating clinical cases and reels being most common in advertisements. Themes also varied significantly, with marketing posts dominating clinical cases and educational content concentrated in procedure discussions.

## Discussion

This study analyzed Instagram posts related to Full Mouth Rehabilitation (FMR) to assess content characteristics and informational accuracy. While Instagram has potential as a tool for public dental communication, the findings revealed considerable variability in content quality, notably dominated by promotional and non-factual posts. This indicates a prioritization of visibility over veracity in health-related social media content, potentially skewing public perceptions regarding advanced dental treatments. A recent systematic review similarly identified widespread misinformation across various health domains, including dentistry, highlighting that nearly 29% of content could be inaccurate or misleading (11).

Only 9% of posts analyzed in this study were educational; however, these consistently aligned with professional standards and contained accurate information. Conversely, 94.5% of promotional posts presented exaggerated or misleading claims, such as “instant smile correction” or “permanent results in a single session,” potentially leading to unrealistic patient expectations. These findings support earlier research indicating that visually appealing but factually questionable content typically gains higher engagement compared to accurate educational material, likely due to emotional appeal and simplicity (12, 13). Despite these risks, promotional content can beneficially increase awareness by visually demonstrating potential outcomes, thus enhancing user understanding of what specific treatments entail and fostering informed curiosity when appropriately moderated. and stimulate public interest in dental procedures by visually showcasing potential aesthetic improvements. To balance engagement with accuracy, promotional materials must incorporate clear disclaimers and contextual information.

The predominance of misinformation emphasizes the ethical implications of social media usage in healthcare, especially when clinical information lacks appropriate qualifications or context. Posts devoid of clinical disclaimers can influence patients toward complex procedures based on superficial visuals rather than informed decisions, underscoring the urgent need for increased transparency, rigorous content moderation, and active professional participation.

Moreover, licensed dental professionals contributed only 5.6% of FMR-related posts, significantly limiting the availability of expert-driven narratives and potentially exacerbating misinformation. Engel et al. (14) noted that patient-generated content often lacks crucial clinical context, leading to the unchecked spread of inaccurate information. Bratland et al. (15) further highlighted concerns regarding anecdotal, non-expert-driven discourse dominating digital health spaces. Although social media presents significant opportunities for healthcare professionals to educate the public, various barriers—including limited time, inadequate digital training, and concerns about maintaining professionalism—often impede their active engagement (5, 16). Addressing these barriers through targeted digital literacy programs and institutional support could encourage dental professionals' proactive participation, significantly improving the credibility and educational quality of online dental health information.

Instagram's inherently visual format greatly enhances user engagement, presenting significant opportunities to promote dental treatments such as FMR effectively. Nevertheless, the prevalent use of before-and-after images can oversimplify complex treatments and lead to unrealistic expectations, particularly when such visuals lack detailed explanations. Thapliyal et al. (17) and Ghahramani et al. (18) have stressed that while Instagram can effectively raise dental health awareness, the absence of expert moderation and structured information substantially diminishes its educational effectiveness. Applying communication theories, such as the Elaboration Likelihood Model (ELM); for example, using visually engaging before-and-after images coupled with comprehensive explanatory captions to encourage deeper audience reflection, can enhance our understanding of these dynamics. ELM posits that while visually attractive promotional content engages users through peripheral cues, educational content that encourages deeper cognitive processing results in better knowledge retention (19).

Engagement metrics further complicate the misinformation landscape, underscoring the need for algorithmic adjustments that prioritize accuracy and reliability over popularity, alongside educational strategies such as digital literacy training programs that teach users to critically evaluate health-related content. on Instagram, as algorithms prioritize content popularity over accuracy, inadvertently amplifying misinformation spread. High engagement with misleading content thus reinforces its visibility and perceived legitimacy. To mitigate these effects, algorithmic moderation mechanisms and educational initiatives aimed at developing users' critical appraisal skills are vital. Such interventions can empower users to discern and evaluate dental health information critically, promoting informed decision-making.

This study has certain limitations, including its cross-sectional design and restriction to English-language posts, potentially limiting content diversity. Future research should explore longitudinal designs, incorporate multilingual content, and directly assess user perceptions to comprehensively understand how Instagram influences public perceptions and decision-making regarding dental treatments. Additionally, examining digital literacy interventions could provide practical insights into reducing susceptibility to misinformation.

Enhancing the credibility of FMR-related content on Instagram requires coordinated efforts among dental professionals, academic institutions, and social media platforms. Recommended strategies include supporting dental professionals to produce engaging, evidence-based content, adjusting algorithms to prioritize accurate health information, implementing robust fact-checking systems, and promoting public digital literacy programs. Such comprehensive initiatives are critical for improving the quality and reliability of oral health communication via social media.

## Conclusion

While Instagram can serve as an educational platform, its current landscape is dominated by promotional content, much of which lacks factual accuracy. Educational posts, though limited in number, were consistently reliable, highlighting the importance of expert participation in online dental communication. Strengthening digital health literacy and promoting evidence-based content are essential steps toward making Instagram a credible resource for advanced dental information.

## Data Availability

The original contributions presented in the study are included in the article/Supplementary Material, further inquiries can be directed to the corresponding author.
